# Supplementary material for: Identification of Freezing-Responsive microRNAs and Their Targets in Chinese Jujube by Small RNA and Degradome Sequencing
Source: Int J Mol Sci. 2026 Sep 4;27(17):7912. doi: 10.3390/ijms27177912 (PMC13566890; doi:10.3390/ijms27177912)
Supplement: Supplementary file 1 [file ijms-27-07912-s001.zip › Supplementary Material S2/novel_miR_138.pdf]

Provisional ID	:	novel_miR_138
Score total	:	0.4
Score for star read(s)	:	-1.3
Score for read counts	:	0
Score for mfe	:	0.1
Score for randfold	:	1.6
Score for cons. seed	:	
Total read count	:	546
Mature read count	:	540
Loop read count	:	0
Star read count	:	6

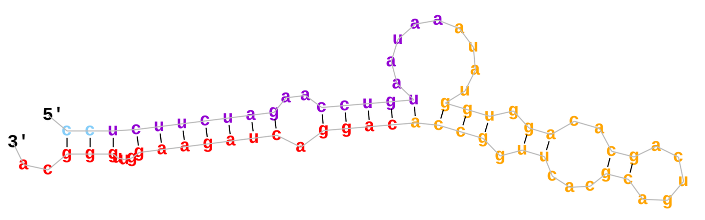

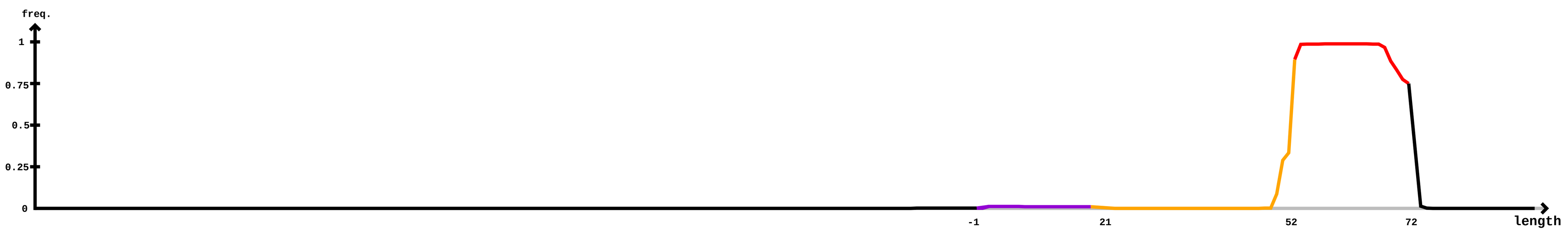

Star Mature

```
5'- uuccguagauauuuuauauguauuugaaaaaaauuuaaauaaaaaauuuugaauggaaucaauaacauuuaucauuuuaaauuaaauauauauauauauauuuuuugaauagauaaauacuguauuaacuccucuaugucuggucguuuaugccugccucuucuagaaccuguaauaaauaugguggacacgacugacgcacuuggccacaggacuagaagguugggcauguauuaacgccuccaugugaa -3'  obs
    uuccguagauauuuuauauguauuugaaaaaaauuuaaauaaaaaauuuugaauggaaucaauaacauuuaucauuuuaaauuaaauauauauauauauauuuuuugaauagauaaauacuguauuaacuccucuaugucuggucguuuaugccugccucuucuagaaccuguaauaaauaugguggacacgacugacgcacuuggccacaggacuagaagguugggcauguauuaacgccuccaugugaa       exp
    ((((((.((.(((((.....((((((((.....)))))))))))))).)).)))))).......((((((..((((((..((((((((....))))))))))))))..)))))))............((((((...((.((((.((((.(((((((((((..(((((.........(((.((..((.....))...)).))))))))).)))))))...))))).)))).))))))..)))).)).  reads  mm  sample
    ..................................................................................................................................................Auuaugccugccucuucu.......................................................................................  1  1  LT-1
    ..............................................................................................................................................................ucuucuCgaaccuguaauaa.........................................................................  1  1  LT-3
    ..............................................................................................................................................................ucuucuCgaaccuguaauaa.........................................................................  4  1  LCK-3
    ..............................................................................................................................................................ucuucuCgaaccuguaauaaa........................................................................  1  1  JCK-2
    ............................................................................................................................................................................................................gCccacaggacuagaagg.............................  1  1  JT-1
    ..............................................................................................................................................................................................................ccacaggacuagaagguu...........................  2  0  LT-1
    ..............................................................................................................................................................................................................ccacaggacuagaagguu...........................  2  0  LT-3
    ..............................................................................................................................................................................................................ccacaggacuagaagguu...........................  8  0  LT-2
    ..............................................................................................................................................................................................................ccacaggacuagaagguug..........................  2  0  LT-3
    ..............................................................................................................................................................................................................ccacaggacuagaagguug..........................  4  0  LCK-3
    ..............................................................................................................................................................................................................ccacaggacuagaagguug..........................  2  0  LT-2
    ..............................................................................................................................................................................................................ccacaggacuagaagguug..........................  1  0  LT-1
    ..............................................................................................................................................................................................................ccacaggacuagaagguuU..........................  2  1  JCK-3
    ..............................................................................................................................................................................................................ccacaggacuagaagguugg.........................  2  0  JCK-3
    ..............................................................................................................................................................................................................ccacaggacuagaagguuggg........................  1  0  LT-3
    ..............................................................................................................................................................................................................ccacaggacuagaagguuggg........................  3  0  LT-1
    ..............................................................................................................................................................................................................ccacaggacuCgaagguugggca......................  1  1  JCK-2
    ..............................................................................................................................................................................................................ccacaggacuagaagguugggca......................  2  0  JCK-3
    ..............................................................................................................................................................................................................ccacaggacuagaagguugggca......................  3  0  LT-3
    ..............................................................................................................................................................................................................ccacaggacuCgaagguugggca......................  1  1  LT-3
    ..............................................................................................................................................................................................................ccacaggacuCgaagguugggca......................  1  1  JCK-3
    ..............................................................................................................................................................................................................ccacaggacuagaagguugggca......................  6  0  JT-1
    ..............................................................................................................................................................................................................ccacaggacAagaagguugggca......................  8  1  LCK-3
    ...............................................................................................................................................................................................................cacaggacuagaagguug..........................  10  0  LCK-3
    ...............................................................................................................................................................................................................cacaggacuagaagguuU..........................  3  1  LT-3
    ...............................................................................................................................................................................................................cacaggacuagaagguuU..........................  1  1  JCK-1
    ...............................................................................................................................................................................................................cacaggacuagaagguug..........................  10  0  JT-1
    ...............................................................................................................................................................................................................cacaggacuagaagguuA..........................  1  1  LT-1
    ...............................................................................................................................................................................................................cacaggacuagaagguug..........................  1  0  JCK-2
    ...............................................................................................................................................................................................................cacaggacuagaagguug..........................  1  0  JCK-1
    ...............................................................................................................................................................................................................cacaggacuagaagguuU..........................  5  1  LT-2
    ...............................................................................................................................................................................................................cacaggacuagaagguug..........................  1  0  LT-3
    ...............................................................................................................................................................................................................cacaggacuUgaagguug..........................  2  1  LCK-3
```

Star Mature

uuccguagauauuuuauauguauuugaaaaaaauuuaaauaaaaauuuugaauggaaucaauaacauuuaucauuuuaaauuaaauauauauauauauauauuuuuugaauagauaaauacuguauuaacuccucuaugucuggucguuuaugccugccucuucuagaaccuguaauaaauauggugg acacgacugacgcacuuggccacaggacuagaagguugggcauguauuaacgccuccaugugaa

...cacaggacuagaagguug...	2	0	LT-2
...cacaggacuagaagguuA...	1	1	LT-3
...cacaggacuagaagguugg...	1	0	JCK-2
...cacaggacuagaagguugg...	2	0	LT-3
...cacaggacuagaagguugg...	1	0	LT-1
...cacaggacuagaagguugg...	6	0	LT-2
...cacaggacuagaagguugg...	5	0	LCK-3
...cacaggacuagaagguugg...	10	0	JT-3
...cacaggacuagaagguugg...	1	0	JCK-3
...cacaggacuagaagguugg...	2	0	JT-2
...cacaggacuagaagguuggg...	1	0	JCK-2
...cacaggacuagaagguuggg...	5	0	JT-2
...cacaggacuagaagguuggg...	1	0	JCK-3
...cacaggacuagaagguuggg...	1	0	LT-3
...cacaggacuagaagguuggg...	5	0	JT-3
...cacaggacuCgaagguugggca...	2	1	LT-3
...cacaggacuagaagguugggca...	2	0	LT-3
...cacaggacuCgaagguugggca...	6	1	JT-2
...cacaggacuagaagguugggca...	1	0	LT-2
...cacaggacuCgaagguugggca...	6	1	JT-3
...cacaggacuagaagguugggca...	1	0	JT-2
...cacaggCcuagaagguugggca...	1	1	LT-2
...cacaggacuCgaagguugggca...	2	1	LT-1
...cacaggacuagaagguugggca...	1	0	JCK-2
...cacaggacuagaagguugggca...	4	0	JT-3
...cacaggacuCgaagguugggca...	13	1	JT-1
...cacaggacuCgaagguugggcau...	2	1	JCK-2
...cacaggacuCgaagguugggcau...	1	1	JCK-3
....acaggacuagaagguugg...	1	0	LT-2
....acaggacuagaagguugg...	1	0	LT-1
....acaggacuagaagguugggca...	6	0	JT-3
....acaggacuagaagguugggca...	5	0	LCK-3
....acaggacuagaagguugggca...	2	0	JCK-3
....acaggacuagaagguugggca...	2	0	LT-1
....acaggacuagaagguugggca...	4	0	JT-2
....acaggacuagaagguugggca...	2	0	JCK-2
....acaggacuCgaagguugggcau...	1	1	JCK-3
....acaggacuCgaagguugggcau...	2	1	JCK-2
....acaggacuagaagguugggcaG...	1	1	LT-3
.....caggacuagaagguuggg...	2	0	LT-2
.....caggacuagaagguuggg...	2	0	LT-1
.....caggacuagaagguuggg...	2	0	LT-3
.....caggacuagaagguuggg...	6	0	JT-3
.....caggacuagaagguuggg...	1	0	JCK-3
.....caggacuagaagguuggg...	4	0	JT-2
.....caggacuagaagguugggc...	4	0	LT-2
.....caggacuagaagguugggc...	2	0	JCK-1
.....caggacuagaagguugggc...	1	0	JCK-2
.....caggacuagaagguugggc...	3	0	JCK-3
.....caggacuagaagguugggc...	1	0	LT-1
.....caggacuagaagguugggc...	1	0	JT-2
.....caggacuagaagguuggAca...	1	1	LT-1
.....caggacuagaagguugggca...	30	0	LT-3
.....caggacuagaagguugggcC...	1	1	JT-1
.....caggacuagaagguugggca...	31	0	LCK-3
.....caggacuagaagguugggca...	31	0	JT-2
.....caggacuCgaagguugggca...	17	1	LT-3
.....caggacuagaagguugggcC...	2	1	LT-2
.....cGggacuagaagguugggca...	4	1	LCK-3
.....caggacuagaagguugggca...	9	0	JCK-2
.....cGggacuagaagguugggca...	5	1	LT-3
.....caggacuCgaagguugggca...	8	1	LT-2
.....caAgacuagaagguugggca...	1	1	LT-1
.....caggacuagaagguugggca...	13	0	JT-1
.....cagAacuagaagguugggca...	2	1	LT-1
.....caggacuCgaagguugggca...	14	1	LCK-3
.....caggacuCgaagguugggca...	14	1	JT-1
.....caggacuagaagguugggca...	1	0	JCK-1
.....caggacuagaagguugggca...	3	0	JCK-3
.....caggacuagaagguugggca...	23	0	LCK-2

Star | Mature

uuccguagauauuuuauauguauuugaaaaaaauuuaaauaaaaauuuugaauggaaucaauaacauuuaucauuuuaaauuaaauauauauauauauauuuuuugaauagauaaauacuguauuaacuccucuaugucuggucguuuaugccugccucuucuagaaccuguaauaaauauggugga cacgacugacgcacuuggccacaggacuagaagguugggcauguauuaacgccuccaugugaa

Read	Count	Mismatch	Library
caggacuagaagguugggca	27	0	JT-3
caggacuagaagguugggca	12	0	LT-2
caggacuCgaagguugggca	1	1	JCK-2
caggacuagaaAguugggca	1	1	JCK-2
caggacuCgaagguugggca	4	1	LT-1
caggacuagaaAguugggca	1	1	JCK-3
caggacuagaagguugggca	7	0	LT-1
caggacuagaagguugggca	25	0	LCK-1
caggacuagaagguugggUa	1	1	LT-3
caggacuagaCgguugggca	1	1	LCK-3
caggacuagaagguuAggca	1	1	LT-3
caggacuagaagguugggcaG	1	1	JCK-3
caggacuCgaagguugggcau	2	1	JCK-3
caggacuCgaagguugggcau	1	1	JCK-1
caggacuagaagguugggcGu	1	1	LT-1
caggacuagaagguugggcaC	1	1	LT-3
caggacuagaagguugggcau	2	0	JCK-2
caggacuagaagguugggcGu	2	1	JCK-3
caggacuCgaagguugggcau	3	1	JCK-2
caggacuagaagguugggcau	2	0	JCK-1
caggacuagaagguugggcau	1	0	LT-3
aggacuagaagguugggc	2	0	LT-3
aggacuagaagguugggc	1	0	JCK-1
aggacuCgaagguugggca	7	1	LT-2
aggacuCgaagguugggca	1	1	LT-1
aggacuagaagguugggca	3	0	LT-3
aggacuagaagguugggca	1	0	LT-1
aggacuCgaagguugggca	1	1	JCK-2
aggacuagaagguugggca	5	0	LT-2
aggacuagaagguugggca	1	0	JCK-2
aggacuCgaagguugggca	1	1	JCK-3
aggacuCgaagguugggca	20	1	JT-1
aggacuCgaagguugggca	4	1	LT-3
aggacuagaagguugggcaAg	7	1	LCK-3
ggacuagaagguugggca	1	0	LT-1
cuagaagguugggcauCu	1	1	JCK-1
